# Plasma-derived extracellular matrix for xenofree and cost-effective organoid modeling for hepatocellular carcinoma

**DOI:** 10.1186/s12967-024-05230-7

**Published:** 2024-05-21

**Authors:** Azza M. El-Derby, Mennatallah A. Khedr, Nehal I. Ghoneim, Mahmoud M. Gabr, Sherry M. Khater, Nagwa El-Badri

**Affiliations:** 1 Center of Excellence for Stem Cells and Regenerative Medicine (CESC), Zewail City of Science and Technology, October Gardens, 6th of October City, Giza, 12582 Egypt; 2 Urology and Nephrology Center, Mansoura University, Mansoura, Egypt

**Keywords:** Hepatocellular carcinoma, Organoids, Extracellular matrix, Platelet-rich plasma, Invasion, Metastasis, Drug resistance, Cancer stem cells, Tumor microenvironment

## Abstract

**Background:**

Hepatocellular carcinoma (HCC) causes significant cancer mortality worldwide. Cancer organoids can serve as useful disease models by high costs, complexity, and contamination risks from animal-derived products and extracellular matrix (ECM) that limit its applications. On the other hand, synthetic ECM alternatives also have limitations in mimicking native biocomplexity. This study explores the development of a physiologically relevant HCC organoid model using plasma-derived extracellular matrix as a scaffold and nutritive biomatrix with different cellularity components to better mimic the heterogenous HCC microenvironment. Plasma-rich platelet is recognized for its elevated levels of growth factors, which can promote cell proliferation. By employing it as a biomatrix for organoid culture there is a potential to enhance the quality and functionality of organoid models for diverse applications in biomedical research and regenerative medicine and to better replicate the heterogeneous microenvironment of HCC.

**Method:**

To generate the liver cancer organoids, HUH-7 hepatoma cells were cultured alone (homogenous model) or with human bone marrow-derived mesenchymal stromal cells and human umbilical vein endothelial cells (heterogeneous model) in plasma-rich platelet extracellular matrix (ECM). The organoids were grown for 14 days and analyzed for cancer properties including cell viability, invasion, stemness, and drug resistance.

**Results:**

HCC organoids were developed comprising HUH-7 hepatoma cells with or without human mesenchymal stromal and endothelial cells in plasma ECM scaffolds. Both homogeneous (HUH-7 only) and heterogeneous (mixed cellularity) organoids displayed viability, cancer hallmarks, and chemoresistance. The heterogeneous organoids showed enhanced invasion potential, cancer stem cell populations, and late-stage HCC genetic signatures versus homogeneous counterparts.

**Conclusion:**

The engineered HCC organoids system offers a clinically relevant and cost-effective model to study liver cancer pathogenesis, stromal interactions, and drug resistance. The plasma ECM-based culture technique could enable standardized and reproducible HCC modeling. It could also provide a promising option for organoid culture and scaling up.

## Background

Hepatocellular carcinoma (HCC) is the most common form of liver cancer. It represented the sixth most diagnosed cancer and the third leading cause of cancer-related death according to the global cancer burden statistics (GLOBOCAN 2020) [1]. Despite advances in therapy, HCC morbidity is rising, with an estimated incidence of over one million cases by 2025 [2]. The onset and progression of cancer are affected by several interconnected factors including the cancer microenvironment, which plays a pivotal role in cancer pathogenesis, progression, metastasis, invasiveness, recurrence, and therapeutic resistance. HCC is a solid tumor that consists of heterogeneous tumor cells and stroma. The HCC tumor microenvironment is recognized for its remarkable inter-tumoral and intra-tumoral heterogeneity that is reflected in the treatment response. The tumor stroma comprises blood and lymphatic vessels, nerves, non-cellular components, and cellular components of non-tumoral cells [3]. The non-cellular components include bioactive substances and the altered extracellular matrix (ECM) that comprises proteins such as collagens, proteoglycans, and the linear glycosaminoglycan hyaluronan. The cellular component is composed of stromal cells, comprising cancer-associated fibroblasts (CAFs), angiogenic cells, inflammatory and immune cells [4]. CAFs originate from a variety of cell types, such as pericytes, endothelial cells (ECs), vascular smooth muscle cells, hepatic stellate cells (HSCs), cancer cells that undergo epithelial-mesenchymal transition (EMT), fibroblasts, and mesenchymal stem/stromal cells (MSCs). MSCs are typically recruited to the injured or hypoxic area within the tumor. The crosstalk between HCC cells and other cells substantially influences tumor cell proliferation, migration, and invasiveness. This cross talk can determine the fate of HCC by promoting vasculogenic mimicry (VM), inhibiting tumor cell apoptosis, activating angiogenesis, and creating an immunosuppressive microenvironment, all of which determine the fate of HCC [5].

Given that intratumoral heterogeneity exists in HCC tumors, the total number of samples needed to be analyzed from a single tumor to reliably represent the tumor microenvironment remains a critical and practical concern [6]. As the interaction between the various cell types in the HCC microenvironment contributes to the main characteristics of cancer, a natural model that mimics the HCC heterogeneous microenvironment is required for a better understanding of liver cancer, and in order to optimize the therapeutic modalities for HCC.

In vitro two-dimensional (2D) cell culture is typically assembled by growing cells on a plastic substrate as an adherent monolayer. The traditional 2D culture of cell lines established from primary liver tumors fails to mimic the diversity and complexity of the tumor microenvironment, and thus is not optimum for studying the cancer microenvironment. Earlier liver “organoids” were engineered by Khaoustov and colleagues using a bioreactor and beads coated with extracellular matrix (ECM) for cell attachment [7]. The group successfully established long-term propagation of a 3D system for liver cells, which were called organoids. The 3D culture system offered an effective model to recapitulate the in vivo architecture of liver cancer and the heterogeneity of the microenvironment [8, 9].

Current HCC organoids are multicellular 3D culture systems that stimulate the cellular and non-cellular components of the tumor. These organoids are composed of a highly proliferative outer region, a middle quiescent region, and a hypoxic core. The extracellular matrix (ECM) contains the biomaterials required to support the organoid structure and mimic the cells’ biochemical and biophysical microenvironment. In addition to its biological functions, the matrix’s physical characteristics, such as stiffness and pliability can alter the biology, morphology, differentiation, and proliferative capacities of the cells [10]. Over the past decades, great advances in organoid engineering have been made thanks to the availability of a myriad of natural and synthetic scaffold materials. Standardization of the protocols however remained challenging due to the many variables in both the cellular and matrix components. For example, batch-to-batch variability of the 3D matrices such as Matrigel represented a challenge in engineering [11, 12]. The residual RNA in the Matrigel also compromised RNA integrity and significantly impacted the resultant gene expression data [13, 14]. The utilization of matrices sourced from mouse tumors also introduced experimental uncertainties and a lack of reproducibility due to their xeno-derived origin [15]. Therefore, there is a growing clinical interest in the use of biomaterials derived from human autologous (or allogeneic) sources to avoid xeno reactions and ensure biodegradability.

Blood is a valuable source of therapeutic material that includes both cellular and protein products [16]. For example, platelet-rich plasma (PRP) and platelet growth factors present in blood have emerged as significant due to their regenerative capabilities. Furthermore, blood-derived biomaterials like platelet-rich fibrin Investigated for their efficacy in promoting healing and tissue regeneration, these components position blood as an invaluable asset in regenerative medicine [16].

Blood biomaterials are biodegradable by endogenous enzymes and rich in nutrients that thwart necrosis and exert valuable physiological advantages [17]. Fibrin sealants (or fibrin glue) were developed in the early 1900s by mixing the blood’s fibrinogen-rich fraction with thrombin and used to stop bleeding and promote wound healing [18]. Platelet gels (PG) are second-generation blood biomaterials that include different types of products based on their mode of preparation and are rich in growth factors and other signaling molecules that promote tissue regeneration [19]. Blood biomaterials are a promising new class of therapeutic materials with a wide range of potential applications, including wound healing, tissue engineering and drug delivery [20]. Their biodegradable nature, rich nutrient content, and ability to promote tissue regeneration make them ideal for a variety of medical applications.

Clinical interest is emerging in platelet growth factor-rich biomaterials (often known as platelet gels or platelet-rich-plasma, PRP). Platelet gels and platelet fibrin glue are rich in platelets that upon thrombin activation, release several growth factors that promote cell growth and differentiation. These blood-derived biomaterials are used increasingly as tissue engineering tools, as they have wide clinical and surgical applications to improve the in vitro or in vivo microenvironment and enhance the success of tissue grafting [16, 21].

Herein, we developed a novel natural, platelet-rich plasma (PRP) scaffold, that promotes cell growth and proliferation in developing organoids. The PRP scaffold is physiologically compatible with human tissues and could be used in vitro to culture functional HCC organoids. We developed a heterogenous organoid that includes a mixture of HCC cells (HUH-7 cell line), a stromal component of BM-MSCs, and an endothelial component of human umbilical vein endothelial cells (HUVEC) in the presence of plasma-derived ECM. This organoid should present a reliable model to study HCC for personalized medicine.

## Materials and methods

### Cell lines and cell culture

HUH-7 cell line and human-bone marrow mesenchymal stem cells (hBM-MSCs, ATCC, USA) were cultured in Dulbecco’s Modified Eagle Medium (DMEM) (Serana, Germany) supplemented with 10% fetal bovine serum (FBS) (Biowest, France), 1% penicillin/streptomycin/amphotericin B (Serana, Germany), and 0.5% L-glutamine (Corning, USA). HUVEC (Thermo Fisher Scientific, USA) were cultured in DMEM/F12 (Biowest, France) supplemented with 2% FBS, 2% penicillin/streptomycin/amphotericin B, 2% L-glutamine, 1 µg/ml dexamethasone (Amrya, Egypt), 250 ng/ml insulin (Acros Organics, USA), 20 µg/ml heparin (Nile, Egypt), 0.025 µg/ml ascorbic acid (Sigma-Aldrich, USA), 5 ng/ml epidermal growth factor (EGF) (Pepro Tech, UK), and 10 ng/ml basic fibroblast growth factor (b-FGF) (Pepro Tech, UK). All the cells were kept at 37 ° C and 5% CO_2_ Nuaire humidified air incubator.

### Scaffold matrix preparation and organoid culture

For PRP preparation, 10 ml of wholeblood was collected from healthy volunteers after securing their informed consent, and following protocol approval by the Institutional Review Boards (IRB) of the Faculty of Medicine at Cairo University and the National Liver Institute at Menoufia University. Blood was added to 80 µl of 25mM of EDTA (Sigma-Aldrich, USA) and centrifuged at 210 g and 4 ° C for 15 min. The upper yellow plasma layer was then transferred to another 15 ml tubeand re-centrifuged at 2600 g and 4 ° C for another 15 min. The upper third layer of the plasma was discarded and the remaining layer was mixed with the platelet pellet and kept at -80 ° C till complete freezing. For the organoid generation, 5 × 10^3^ cells of HUH-7 alone (3D HUH-7 group), or a heterogeneous combination of HUH-7, HUVEC, and hBM-MSCs at a ratio of 10:7:2 (3D Mixed group) were suspended in 50 µl of 50% PRP, 45% high glucose DMEM (Serana, Germany), and 5% of 3% CaCl_2_ solution (Alpha Chemika, India), and cultured in a dome shape in 24 well plates.

The starting seeding density of the organoids was maintained equally among the different groups; The difference between the groups was in the cellular composition only, while the organoid volume was fixed, to avoid misleading results due to size variations. Plates were kept at 37 ° C for 30 min until complete ECM solidification, then covered with high glucose DMEM supplemented with 10% FBS, 1% penicillin/streptomycin/amphotericin B, and 0.5% L-glutamine, or 1:1 of HUH-7 culture medium and HUVEC culture medium, and kept at 37 ° C and 5% CO2 humidified air incubator for 14 days. The culture medium was replenished every 3–4 days. The organoid groups (3D HUH-7 and 3D Mixed) were tested compared to 2D cultured HUH-7 cells (HUH-7 2D) and HCC tissues (HCC tissue) throughout the study.

### Real-time qPCR

mRNA was extracted from organoids, cells and tissues using TRIzol reagent (Thermo Fisher Scientific, USA) according to the manufacturer’s protocol and quantified for the concentration and purity by Thermo Scientific™ NanoDrop™ 2000/2000c Spectrophotometer. cDNA was synthesized using Revert Aid First Strand cDNA Synthesis Kit (Thermo Fisher Scientific, USA) according to the manufacturer’s protocols. Real-time qPCR was performed using HERA PLUS qPCR SYPER Green kit (Willowfort, UK). The primers’ sequences are listed in Table 1. The relative gene expression was calculated using the 2^-ΔΔct^ method, $\beta$-actin gene was used for normalization and each reaction was performed in triplicates.

**Table 1:** Sequence of the primers used

Name	F/*R*	5` - Oligo Seq − 3`	Name	F/*R*	5` - Oligo Seq − 3`
CCNA1	F	GCACACTCAAGTCAGACCTGCA	EPCAM	F	CGCAGCTCAGGAAGAATGTG
	R	ATCACATCTGTGCCAAGACTGGA		R	TGAAGTACACTGGCATTGACG
CCNB1	F	GACCTGTGTCAGGCTTTCTCTG	ABCG2	F	TTTCCAAGCGTTCATTCAAAAA
	R	GGTATTTTGGTCTGACTGCTTGC		R	TACGACTGTGACAATGATCTGAGC
CCND2	F	GCTGTCTCTGATCCGCAAGC	CA9	F	GTGCCTATGAGCAGTTGCTGTC
	R	AGGGCATCACAAGTGAGCGA		R	AAGTAGCGGCTGAAGTCAGAGG
CCNE1	F	TGTGTCCTGGATGTTGACTGCC	WNT7B	F	CCTCCCTGGATCATGCACAG
	R	CTCTATGTCGCACCACTGATACC		R	CACGTACAGGACGCCAAAGC
APAF1	F	GCCAAGCAGGAGGTCGATAATG	APOBEC3B	F	GCGCCAGACCTACTTGTGCT
	R	GACCATCCTCAGAAAAGCAGGC		R	CCGGGTCCAACTCGTTGCATAG
B-actin	F	AGAGCTACGAGCTGCCTGAC	DLG5	F	TCAGCAGTGTGGGCACTACC
	R	AGCACTGTGTTGGCGTACAG		R	AAAGGCCGTGCCATGCGTAG
TP53	F	GTTCCGAGAGCTGAATGAGG	APOBEC3B	F	GCGCCAGACCTACTTGTGCT
	R	TTATGGCGGGAGGTAGACTG		R	CCGGGTCCAACTCGTTGCATAG
BAX	F	CAAACTGGTGCTCAAGGCCC	GABRD	F	ACCACACCAACGAGACCCTG
	R	GAGACAGGGACATCAGTCGC		R	GCAGCCGGATGAGCTTGTTC
BCL2	F	GGATAACGGAGGCTGGGATG	FAM186A	F	AACCCACGCTTTGGAGTCCC
	R	TGACTTCACTTGTGGCCCAG		R	TTGCTTAAGGGTGAGGGGCG
MMP-2	F	TTCACCCACATCAGGAACCCC	TGF-α	F	CGCCCGTAAAATGGTCCCCT
	R	ACTGCCTTCGATACACCGGG		R	GGCACGCAGCCAACACAATA
MMP-3	F	CACTCACAGACCTGACTCGGTT	IGF2	F	TGGCATCGTTGAGGAGTGCTGT
	R	AAGCAGGATCACAGTTGGCTGG		R	ACGGGGTATCTGGGGAAGTTGT
MMP-13	F	TTCGGCTTAGAGGTGACTGGC	TNFSF10	F	TGGCAACTCCGTCAGCTCGTTA
	R	TTCACCCACATCAGGAACCCC		R	AGCTGCTACTCTCTGAGGACCT
E-cadherin	F	GTCACTGACACCAACGATAATCCT	KRAS	F	TGTTCACAAAGGTTTTGTCTCC
	R	TTTCAGTGTGGTGATTACGACGTTA		R	CCTTATAATAGTTTCCATTGCCTTG
SNAIL	F	ACCACTATGCCGCGCTCTT	CD24	F	TGCTCCTACCCACGCAGATT
	R	GGTCGTAGGGCTGCTGGAA		R	GGCCAACCCAGAGTTGGAA
Vimentin	F	TGTCCAAATCGATGTGGATGTTTC	CD44	F	AGAAGGTGTGGGCAGAAGAA
	R	TTGTACCATTCTTCTGCCTCCTG		R	AAATGCACCATTTCCTGAGA
AFP	F	AGCAGCTTGTTAAATCAACATGCA	RHOA	F	GGCAAACAGGATTGGCGCTT
	R	AAAATTAACTTTGGTAAACTTCTGACTCAGT		R	CCGCATAAGGGCTGTGCTTG
TGFB	F	CAGCAACAATTCCTGGCGATA	P21	F	TGGAACTTCGACTTTGTCAC
	R	AAGGCGAAAGCCCTCAATTT		R	CACATGGTCTTCCTCTGCT
TCF4	F	GCCTCTTCACAGTAGTGCCATG	C-MYC	F	AAACACAAACTTGAACAGCTAC
	R	GCTGGTTTGGAGGAAGGATAGC		R	ATTTGAGGCAGTTTACATTATGG
CD133	F	CAGAGTACAACGCCAAACCA			
	R	AAATCACGATGAGGGTCAGC			

### MTT assay

MTT assay is routinely used for viability analysis of the 2D culture. Cells were treated with 5 mg MTT powder (SERVA, Germany) in 1 ml 1x phosphate buffer saline (PBS) (Loba Chemie, India) and incubated for 3 h at 5% CO_2_ Nuaire humidified air incubator at 37 ° C. The formed formazan salts were dissolved by using dimethyl sulfoxide (DMSO) (Serva, Germany) for 15 min with continuous shaking. The optical density was measured at 570 nm using a FLUOstar Omega microplate reader.

### Calcein AM viability staining

Calcine AM is used in 3D cultures to determine the viability of the whole construct without disturbing it or the matrix. The organoids were washed twice with PBS for 5 min each at 37 ° C. 100 µl of 0.25 µM Calcein AM staining solution (Life Technologies, USA) was added to the organoids or to cells and incubated at 37 ° C for 10 min, after which the green staining solution was removed and organoids or cells were washed twice with PBS for 5 min at 37 ° C. The images were captured using Leica inverted fluorescent microscope and image analysis was performed using ImageJ 1.53 K software.

### Cell viability analysis with flow cytometry

Flow cytometry analysis was used to obtain a quantified ratio of viable cells. Organoids were washed with 1x PBS for 10–15 min then collected using 1.5% trypsin and incubated at 37 ° C to fully dissolve the scaffold. A complete culture medium (CCM) was added, and the cell suspension was collected and centrifuged at 2000 RPM at 15 ° C for 10 min. After discarding the supernatant, the cell pellet was washed twice with 1x PBS and centrifuged at 300 x g for 5 min. The pellet was then re-suspended in 200 µl FACS buffer with 10 µl of 50 µg/ml PI and transferred into 15 ml tubes (Becton Dickinson, USA) for 15 min incubation in the dark at room temperature. Cell viability was assessed by fluorometric measuring of the concentration of intracellular PI in the cells using flow cytometer Becton Dickinson (BD, USA) and data analyzed by FlowJo v. 10.2 software (BD Life Sciences).

### Cryosectioning

Organoids were washed 3x with D-PBS for 10 min. each, then 4% paraformaldehyde (PFA) solution was added and incubated overnight at 2–8 ° C. The organoids were washed three times again for 10 min each with PBS-Tween (1000:1), and incubated with 30% sucrose solution overnight at 2–8 ° C. The sucrose solution was removed and a solution of 7.5% gelatin dissolved in 10% sucrose was added and the organoids were incubated at 37 ° C for one hour. Finally, the organoids were transferred to the embedding molds then added to a cold bath of dry ice/100% ethanol and transferred to -80 ° C freezer. The samples were then embedded in a tissue-tek O.C.T, and cut using cryomicrotome (Leica, USA) into 5-µm-thick sections on a positively charged slides for further staining.

### Immunofluorescence staining

The organoids were washed 3x with 1x PBS/10 minutes and permeabilized with 0.3% Triton x-100 (Sigma-Aldrich, USA) at room temperature for 15 min, followed by an additional washing cycle using 1x PBS, and bovine serum albumin (BSA) (Sigma-Aldrich, USA, 5%) for 2 h at room temperature. Anti-Hu CD326 (EpCAM) (Invitrogen, USA) and cytokeratin19 monoclonal antibodies (Invitrogen, USA) were diluted in blocking solution to the recommended concentration according to the manufacturer’s instructions and incubated with the organoids for 16 h at 2–8 ° C in the dark. The organoids were washed 3x with 1x PBS/10 minutes. Alexa Fluor 488 donkey anti-mouse IgG (Life Technologies; USA) was diluted with blocking solution to the recommended concentration according to the manufacturer’s instructions, followed by incubation with the organoids for 2 h at room temperature in a dark humidified chamber. The organoids were washed with 1x PBS 3x for 10 min each. 3 µg/ml Hoechst 33,342 (Life Technologies, USA) as a counterstain was added for 15 min at room temperature in a dark humidified chamber. The organoids were washed again and visualized using Leica inverted-fluorescent microscope and data was analyzed using ImageJ 1.53 K software.

### Cancer stem cell marker analysis

Organoids were washed with 1x PBS for 10–15 min then incubated in 1.5% trypsin at 37 ° C for 20–30 min. The cell suspension was centrifuged at 2000 RPM at 15 ° C for 10 min, and the pellet was washed with 1x PBS and centrifuged at 2000 RPM at 15 ° C for 10 min. The cell pellet was re-suspended in 200 µl FACS buffer and distributed equally in two 5mL round bottom polystyrene FACS tubes. The cells in each tube were stained with 3 µl FITC-conjugated anti-CD44 and PE-conjugated anti-CD24 (BD Biosciences, USA), and then incubated in a dark humidified chamber at room temperature for 30 min. The cells were suspended and centrifuged at 2000 RPM at 15 ° C for 10 min followed by re-suspending the pellet in 300 µl FACS buffer. Fluorescence was measured using BD flow cytometer; the data was analyzed using FlowJo v. 10.2 software.

### Side population assay

The organoids were washed with 1x PBS for 10–15 min and incubated with 1.5% trypsin at 37 °C for 20–30 min till complete dissolving, followed by centrifugation at 2000 RPM at 15 ° C for 10 min. The cell pellet was re-suspended in CCM with 2% FBS into single-cell suspension. The cells were stained with 5 µg/ml Hoechst 33,342 (Life Technologies, USA) for 90 min at 37 ° C. The cell samples were analyzed using a flow cytometer with VL-1 A filter; the data was analyzed using FlowJo v. 10.2 software.

### Scanning electron microscopy (SEM)

The organoids were fixed using 2.5% glutaraldehyde at 4 ° C for 1 h, followed by a dehydration process through removing 2.5% glutaraldehyde and adding graded 70%, 80%, and 95% ethanol for 30 min each, then changing into 100% ethanol 3 times for 45 min each. Subsequently, the organoids were subjected to freeze drying, and their extracellular matrix fiber architecture was observed using scanning electron microscopy (SEM). Surface images of the organoids were recorded using Neoscope (JCM-6000 Plus) JEOL Benchtop SEM (JEOLCompany, Tokyo). Samples were mounted onto SEM stubs. Applied SEM conditions were a 10 mm working distance, with an in-lens detector with an excitation voltage of 10 kV.

### Enzyme-linked immunosorbent assay (ELISA)

The assay was performed using an ELISA kit for the analysis of human AFP (Elabscience, USA) according to the manufacturer’s instructions. Briefly, 100 µl of each of the standard medium and the culture medium surrounding the organoids (sample) were added to the wells and incubated for 90 min at 37 ° C. The standard and the samples were discarded and 100 µl biotinylated detection Ab working solution was added to each well and incubated for 60 min at 37 ° C. The wells were washed 3 times followed by the addition of 100µL HRP conjugate working solution and incubated for 30 min at 37 ° C. The solution was discarded, and the wells washed 5 times. 90 µl of substrate reagent was added immediately and incubated for 15 min at 37 ° C. 50 µl of stop solution was added and the plate was read directly at 450 nm using a FLUOstar Omega microplate reader.

### Xenotransplantation of organoids in nude mice

The animal experiments were performed at the Urology and Nephrology Center Animal House according to the guidelines of the institutional and National Institute of Health for the care and use of animals in the laboratory. The study design and protocol were approved by the Animal Ethics Committee of Mansoura University. Two groups of 3 mice each (Swiss Nude Nu/Nu mice, Charles River Laboratories, Paris, France) were anesthetized using ketamine (100 mg/kg) and diazepam (5 mg/kg). Eight organoids collected at day 14 were implanted in each mouse subcutaneously. The mice were euthanized after one month, and subcutaneous sections were stained for histological analysis.

### Statistical analysis

The data were presented as mean ± SD. Significance was calculated using one-way ANOVA and two tailed T-test. *p* < 0.05 was considered statistically significant.

## Results

### Human plasma-derived nutritive ECM scaffold maintains cells viability and proliferation

**Fig. 1:**
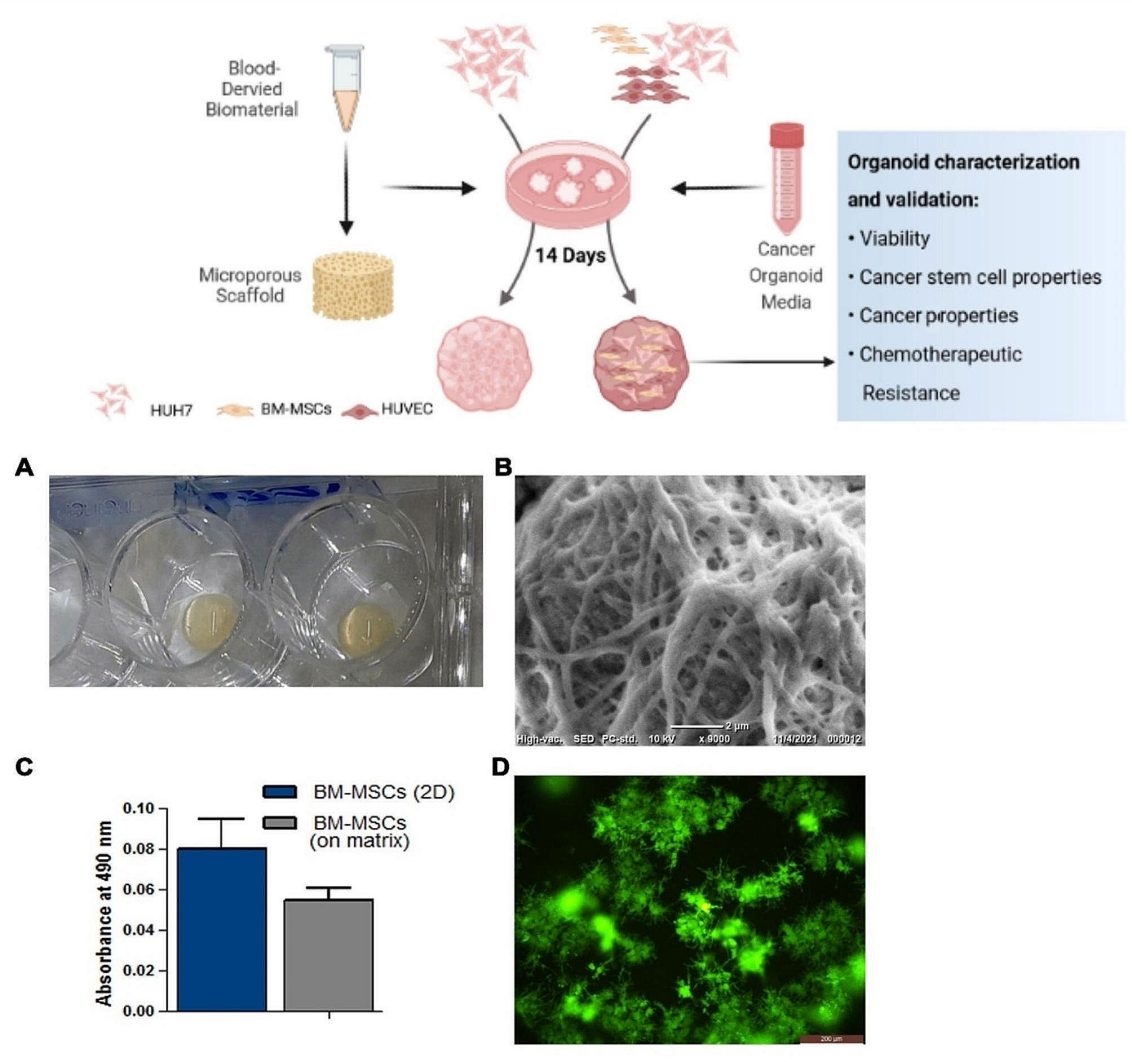
Plasma-derived ECM characterization. **(A)** Light photograph of the plasma-derived biomatrix applied in organoid culture. **(B)** Scanning electron microscopy (SEM) micrograph of extracellular matrix fiber architecture. **(C)** MTT assay for BM-MSCs cultured with and without the ECM for 72 h. **(D)** Cell viability was confirmed using Calcein AM staining after 3 days of culture

Human plasma was polymerized in the presence of calcium to formulate 50 µl dome-shaped drops in 24 well plates for organoid culture (Fig. 1A). The plasma ECM ultra-structure (Fig. 1B) displayed a porous mesh network-like structure suitable for cell growth and 3D self-assembly. To confirm that the matrix can sustain cell viability, the viability of the BM-MSCs was assessed. BMSCs were seeded at a density of 50 K in ECM-coated and non-coated wells, and supplemented with the appropriate culture medium for 72 h before the MTT assay was conducted as mentioned in the Methods section (Fig. 1C). Compared to cells cultured on regular 2 D culture plates, no significant change was observed in cell viability on the scaffold as shown by bright green Calcein AM staining (Fig. 1D).

### Plasma-derived ECM and stromal cell compartments supported HCC organoid growth and assembly

**Fig. 2:**
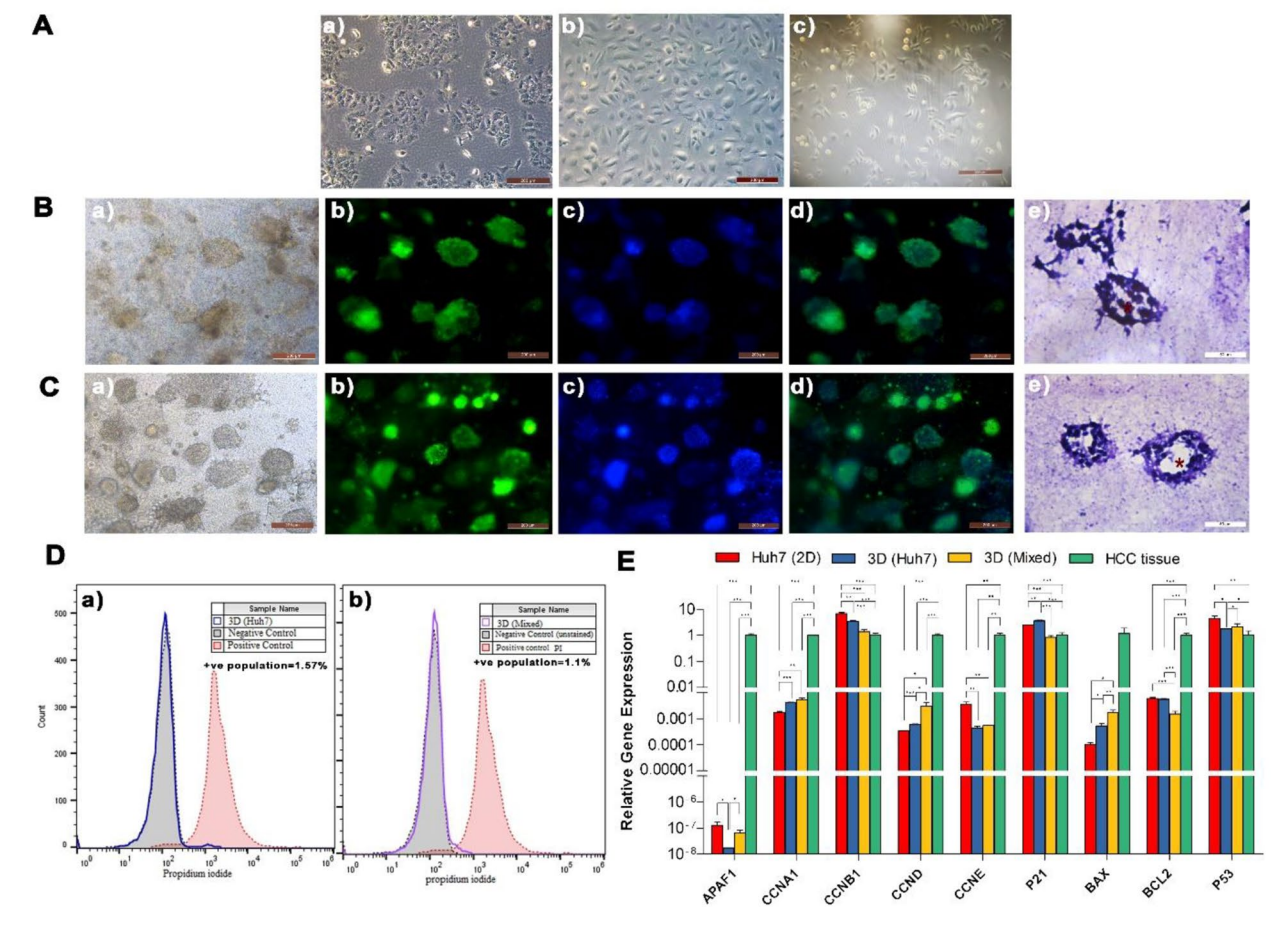
**A**. Phase-contrast micrograph of the HUH-7, HUVEC, and BM-MSCs cells (a-c). **B.** Micrograph of 3D HUH-7 organoids and **C**. 3D Mixed organoids, (a) stained with Calcine AM (b) Hoechst (c) and merged (d) H & E stained cryosections show the structure and lumen (*) of the 3D Mixed organoids surrounded by a network of stromal cells (e). **D.** Flow cytometry analysis of cell viability using propidium iodide staining. (a) HUH-7 organoids (b) 3D Mixed organoids after 14 days. **E.** RT-qPCR shows relative levels of expression of cell cycle regulator (CCNA1, CCNB1, CCND, and CCNE), proliferation (BAX, BCL2), and apoptosis genes (P21, P53, APAF1) in HUH-7 2D, 3D HUH-7, 3D Mixed, and HCC tissue. Values represent relative gene expression mean ± SD. ^*^p < 0.05, ^**^p < 0.01, and ^***^p < 0.001 respectively

The plasma-derived ECM was used to support the growth of two types of HCC liver organoids. The first was engineered using a single cellular component, HUH-7 cell line (3D HUH-7 organoid), and the second by coculture of HUH-7 cells with BM-MSCs and HUVECs for 14 days (3D Mixed) (Fig. 2A). Organoids started to aggregate and self-assemble from day 7 and were formulated by day 12–14 (Fig. 2Ba). The viability of the generated constructs was tested by Calcein AM staining (Fig. 2B. b, c). On day 14, organoids were collected, and cells dissociated and tested for viability using propidium iodide staining. Flow cytometry analysis showed 98.43% and 98.9% viability in 3D HUH-7, and 3D Mixed organoids respectively indicating sustained viability in both constructs (Fig. 2D).

### Diminished apoptosis and improved progression in HCC organoids

Our data show significant upregulation in cell regulators cyclin A (CCNA1) and cyclin D (CCND) in both 3D HUH-7 and 3D Mixed organoids compared to HUH-7 cells (*p* < 0.05). However, both were lower than their counterparts in primary HCC tissue. Cyclins B (CCNB1) and E (CCNE) were significantly downregulated (*p* < 0.01) in the 3D HUH-7 and 3D Mixed organoids compared to HUH-7 2D cells (Fig. 2E). HUH-7-derived organoids showed significant downregulation in apoptosis markers AFAP1 and P53 (*p* < 0.05), and significant upregulation in P21 and BAX (*p* < 0.01, *p* < 0.05 respectively) compared to HUH-7 cells. 3D Mixed organoids showed significant downregulation in APAF1, P21, and BCL2 (*p* < 0.05) and upregulation in BAX compared to HUH-7 cells (*p* < 0.05). 3D Mixed organoids also showed more similar expression patterns of APAF1, CCNA1, CCNB1, CCND, CCNE, P21, BAX, and P53 to the HCC tissue than 3D HUH-7 organoids as shown in (Fig. 2E).

### Augmented cancer properties of HUH-7 in the homogenous and heterogenous organotypic culture

**Fig. 3:**
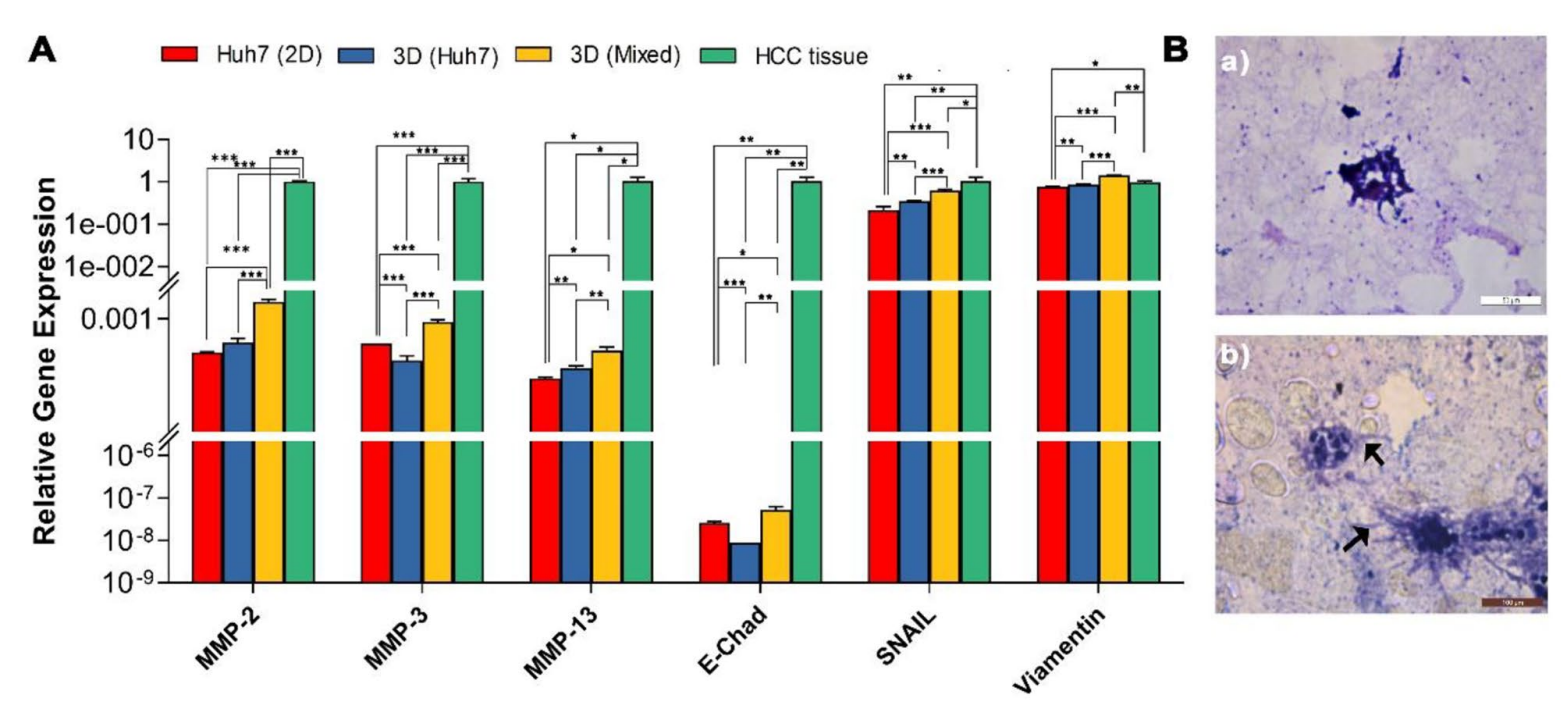
Invasion potential. **(A)** Gene expression of EMT markers and invasion markers in HUH-7 2D), organoids (3D HUH-7 and 3D Mixed organoid groups, and HCC tissue. Values represent relative gene expression mean ± SD. ^*^p < 0.05, ^**^p < 0.01, and ^***^p < 0.001 respectively. **(B)** H&E stained cryosections micrograph of (a) 3D HUH-7 (b) 3D Mixed organoid groups after 14 days show protrusive organoid structure with cytoplasmic filaments (arrow) invading the biomatrix

To evaluate the effect of including stromal cells component on enhancing the cancer-related.

characteristics of the organoids, similar to HCC tissue a significant upregulation in the expression of the invasion metalloproteases MMP-2, MMP-3, and MMP-13 were noticed in 3D (Mixed ) organoids compared to the 2D cultured HUH-7 cells (HUH7(2D)) and 3D (HUH-7). (Fig. 3A). Also the EMT markers, E-cadherin, Snail and Vimentin expression levels were markedly increased in 3D Mixed group compared to HUH-7 2D cultured cells (*p* < 0.001,0.001.0.05,0.05,0.01,0.01) and 3D HUH-7 organoids (*p* < 0.001, 0.001.0.01, 0.01,0.001,0.001 respectively) (Fig. 3A). Invasion markers were further confirmed by prominent cytoplasmic filaments invasion of the ECM in the cryosections of 3D HUH-7 (Fig. 3Ba) and 3D Mixed organoids (Fig. 3 Bb).

**Fig. 4:**
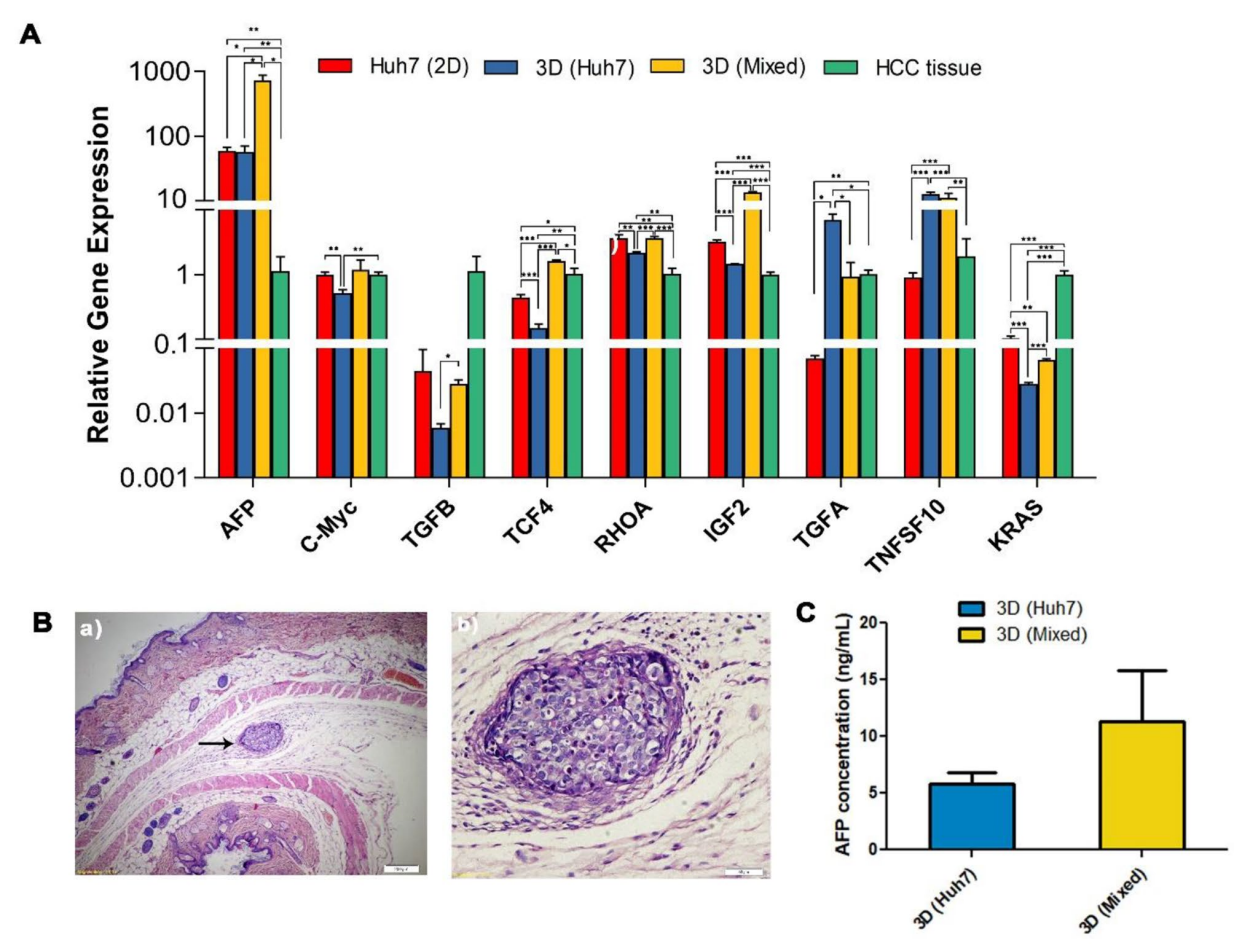
HCC marker expression in the organoids. **(A)** Gene expression of HCC markers in HUH-7 2D, 3D HUH-7, and 3D Mixed organoid groups, and HCC tissue. Values represent relative gene expression (mean ± SD). ^*^p < 0.05, ^**^p < 0.01, and ^***^p < 0.001 respectively. **(B)** Micrograph of H&E-stained skin tissue of Nude mice one month after subcutaneous transplantation of the 3D HUH-7 organoid. (a) The hypodermis shows a nested sheet of malignant cells (arrow) with few apoptotic cells amidst adipocytes and hair follicles with scattered capillaries. The surrounding stroma shows scattered inflammatory cells, fibroblasts, and edema (b). **(C)** Alpha-fetoprotein concentration level in the culture supernatant of 3D HUH-7 and 3D Mixed organoids at day14

Analysis of the HCC markers showed significant upregulation in AFP genes (*p* < 0.05) and protein (Fig. 4C) levels in 3D Mixed compared with 3D HUH-7 organoids. Results also showed significant upregulation of c-Myc, TGF$\beta$, TCF4, RHOA, and IGF2 gene expression in (3D Mixed) organoid group compared with both the HUH-7 cell line and 3D HUH-7 organoids. On the other hand, 3D HUH-7 organoids showed significant downregulation in c-Myc, TGF$\beta$, TCF4, RHOA, IGF2, and KRAS gene expression level compared to 2D HUH-7 cell culture. Also, a significant increase in TGF-$\alpha$ expression in 3D HUH-7 organoids was observed compared to the 3D Mixed organoids counterpart. To determine the tumor-forming potential of HUH-7 organoids cultured in ECM, day 14 organoids collected from 3D HUH-7 and 3D Mixed groups were transplanted subcutaneously in Nude mice. After 4 weeks, the mice were euthanized, and skin was collected for histological analysis. Histological sections staining with H&E showed that the 3D HUH-7 group formed a nested sheet of malignant cells with few apoptotic cells amidst adipocytes and hair follicles with scattered capillaries in the hypodermis. The malignant sheet was surrounded by stroma along with scattered inflammatory cells, fibroblasts, and edema (Fig. 4B). In the 3D Mixed organoid group, the epidermis showed intact layers but of less thickness. The dermis showed fine dermal papillae and the reticular layer showed intact acellular components of the extracellular matrix in addition to a few fibroblasts and scattered capillaries. The hypodermis showed mature adipocytes and hair follicles with scattered capillaries indicating no tumor formation.

### Cancer stem cell and drug resistance in HCC organoids

**Fig. 5:**
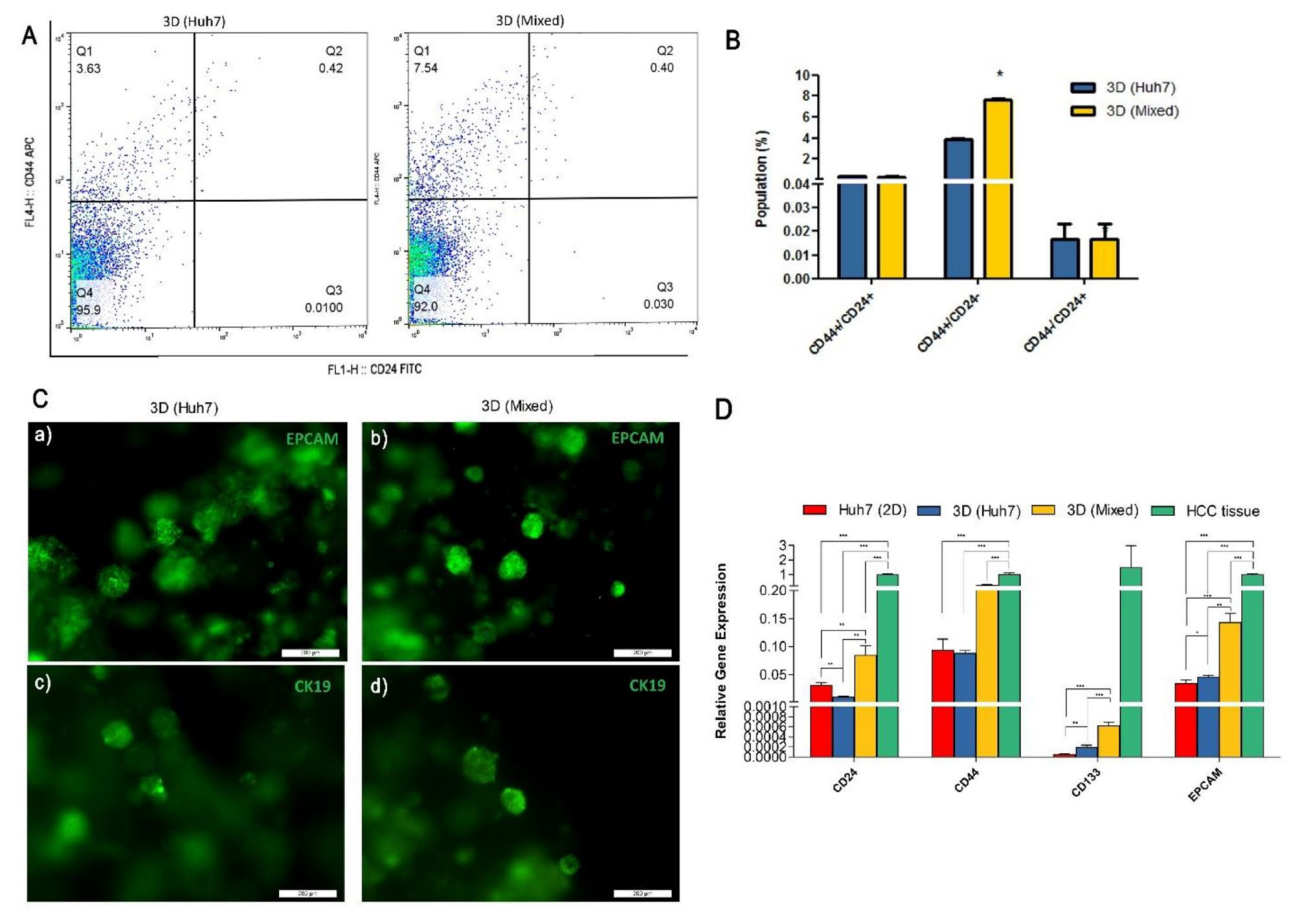
CSC markers. **A, B.** Flow cytometry analysis of CD44^+^/CD24^+^ cell population shows significantly higher CD44^+^ population in the 3D Mixed compared to 3D HUH-7 organoids. **C.** Fluorescent micrographs for the organoids generated from day 14. 3D HUH-7 and 3D Mixed organoids stained for EPCAM (a, b) and CK19 (c, d). **D.** Gene expression analysis of CSC markers in 2D cultured HUH-7 cells, 3D HUH-7, 3D Mixed organoids, and HCC tissue. Values represent relative gene expression mean ± SD. ^*^p < 0.05, ^**^p < 0.01, and ^***^p < 0.001 respectively

We evaluated the cancer stem cell markers in the organoids to determine the potential of the developed model to be used for chemoresistance studies. Flow cytometry analysis of CD44 and CD24 showed a significant increase in the CD44^+^CD24^+^ population in the 3D Mixed organoids group compared to 3D HUH-7 organoids (*p* < 0.05) (Fig. 5A-B) [22]. EPCAM and cytokeratin 19 were reported as progenitor and tumor stem cell markers in HCC [23]. CK19-positive HCCs demonstrated aggressive behavior and poor outcome [24, 25]. Immunofluorescence analysis showed positive staining for EPCAM (Fig. 5C-a, b) and CK 19 (Fig. 5C-c, d) with a more intense signal from the 3D Mixed compared to the 3D HUH-7 organoids. Significant upregulation in the gene expression level of tumor stem cell markers CD24, CD44, CD133, and EPCAM were also observed in the 3D Mixed organoids compared to both HUH-7 cell line and 3D HUH-7 organoids. Gene expression patterns were similar to the HCC tissue organoids as shown in Fig. 5D.

**Fig. 6:**
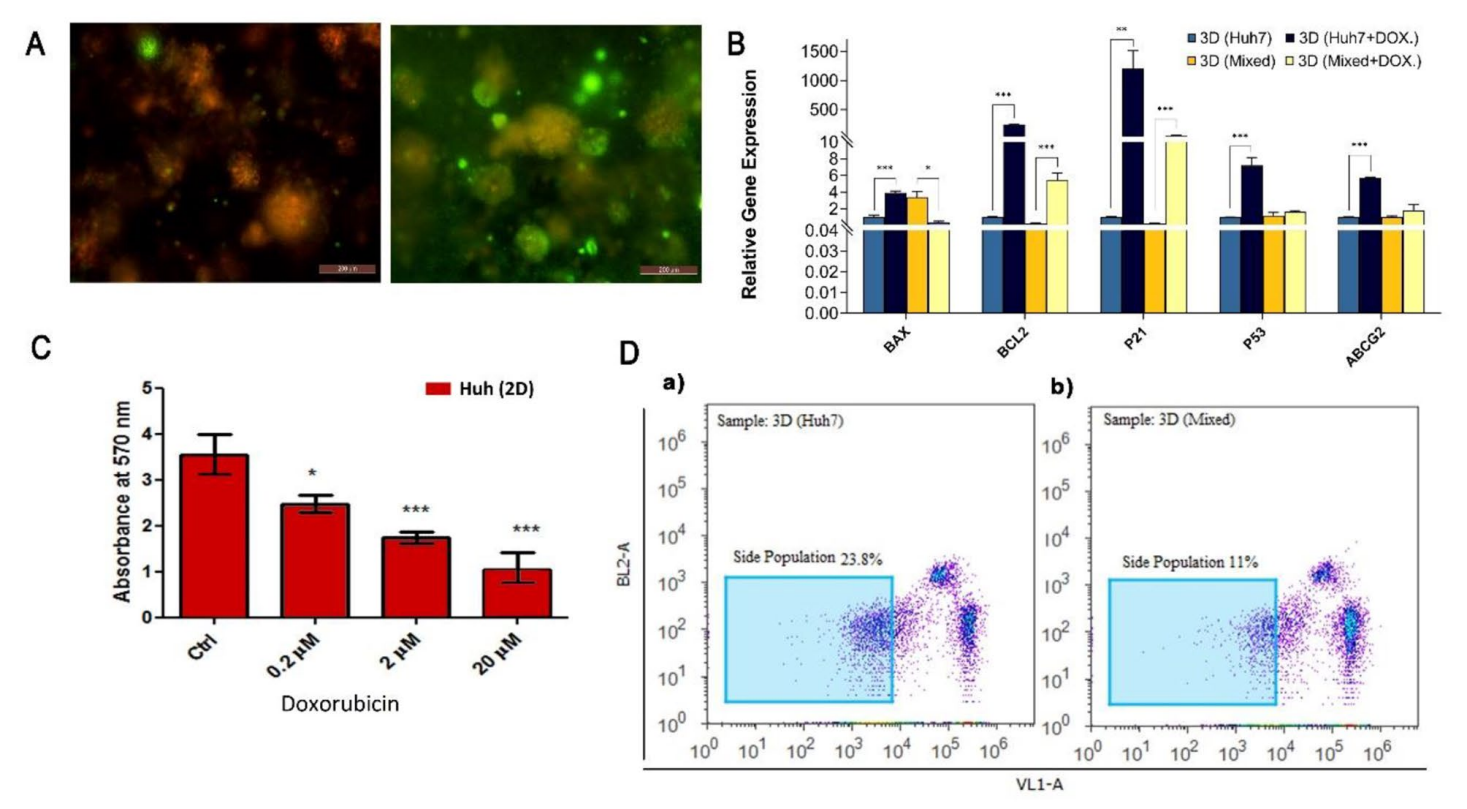
Chemotherapy resistance. A. Fluorescent micrograph of 3D HUH-7 (a) and 3D Mixed organoids (b) stained with Calcein AM after treatment with Doxorubicin (2 μm) for 72 h. **B.** Gene expression analysis of proliferation (BCL2, BAX), apoptosis (P53, P21), and drug resistance (ABCG2) markers in HUH-7 cells cultured in HUH-7 2D, 3D HUH-7, 3D Mixed organoid, and HCC tissue. **C.** MTT assay for HUH-7 cells treated with various concentrations of doxorubicin for 72 h. For figures B and C, values represent the mean of absorbance ± SD. ^*^p < 0.05, ^**^p < 0.01, and ^***^p < 0.001 respectively. **D.** Identification of side population (SP) cells from 3D HUH-7 (a) and the 3D Mixed organoids (b)

We tested the organoid’s response to doxorubicin chemotherapy [26]. Calcein AM staining was used to determine the viability of the whole construct without disturbing it after being exposed to the same dose and treatment time of the chemotherapy [27]. Doxorubicin was one of the earliest chemotherapeutic drugs used for HCC treatment with confirmed results [28, 29]. It exerts a wide range of effects on both tumor and non-tumor cells through reactive oxygen species (ROS) generation, inhibition of DNA synthesis, and DNA damage [30]. Moreover, it has been reported that doxorubicin treatment can induce the cancer stem cell population in HCC and increase their number compared to non-treated groups [31]. Doxorubicin was used as a good example of chemotherapeutic treatment for HCC to test our organoid model and explore the potential cancer stem cell and resistance that could be developed in the heterogeneous model. After 3 days of treatment, 3D Mixed organoids maintained more viability compared to 3D HUH-7 ones as shown by the Calcein AM stain (Fig. 6A). Significant upregulation of apoptosis related genes, BAX (*p* < 0.001), BCL2 (*p* < 0.001), P21(*p* < 0.01), P53 (*p* < 0.001), and ABCG2 (*P* < 0.001) was reported in 3D HUH-7 organoids after treatment compared to the untreated group. On the other hand, 3D Mixed organoids showed significant upregulation in p21 (*p* < 0.001) and BCL2 (*p* < 0.001), and downregulation in BAX (*p* < 0.05) after treatment compared to the untreated control. Hoechst-stained cells sorted by flow cytometry showed side population (SP) that excludes Hoechst 33,342 dye (DNA binding dye) of about 11% and 23% of 3D Mixed and 3D HUH-7 organoids respectively (Fig. 6D).

### Molecular signature of the generated HCC organoids

**Fig. 7:**
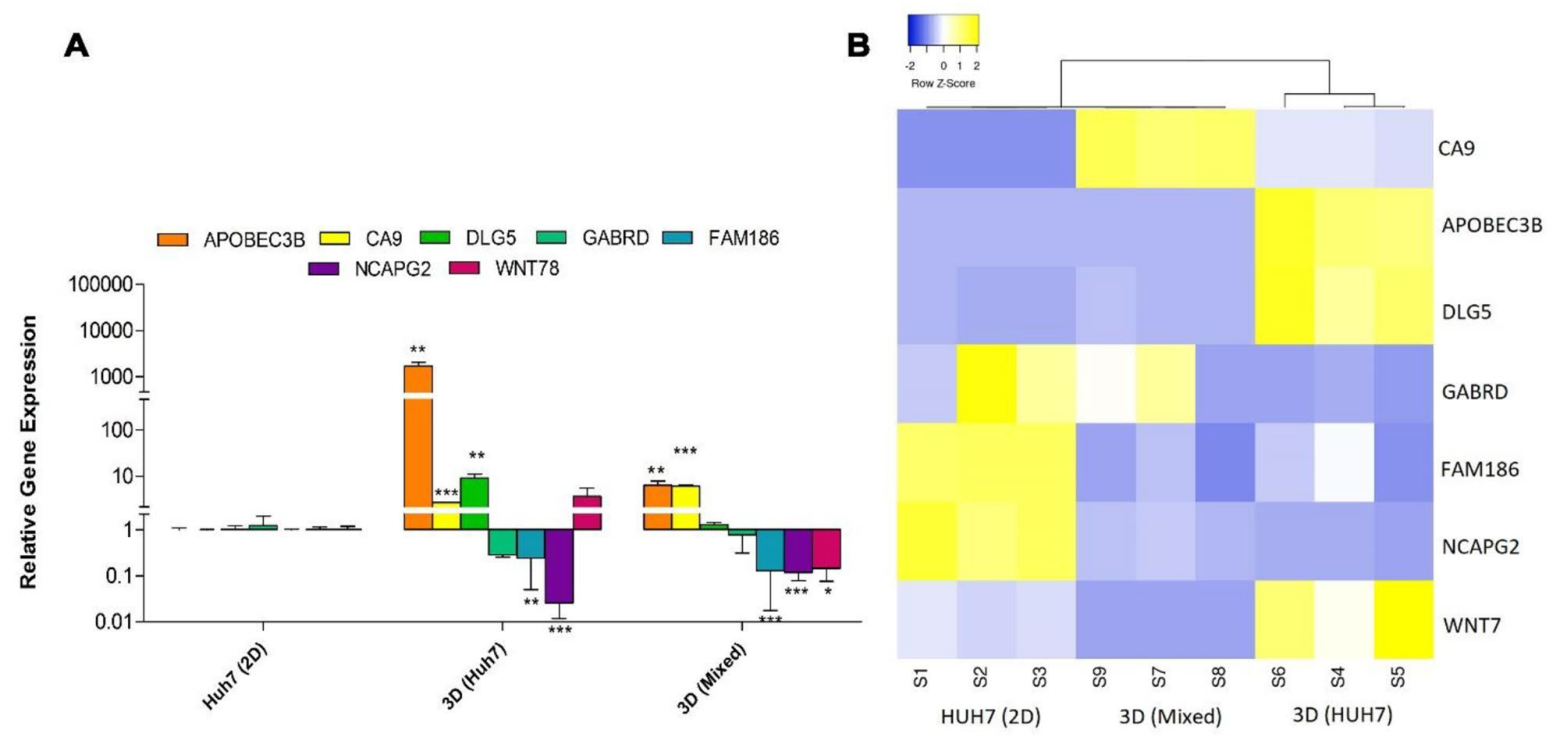
Molecular signature. **(A)** Heatmap of differentially expressed genes in different HCC stages. Genes detected by quantitative reverse transcription PCR (RT-qPCR). The rows represent genes, and the columns represent replicates of 3D HUH-7, 3D Mixed organoids, compared to HUH-7 2D cells. Blue indicates “downregulation” and yellow indicates “upregulation”. The clustering is done using the complete-linkage method together with the Euclidean distance measure. **(B)** The corresponding gene expression analysis of the same panel of genes represented by a bar chart of HUH-7 cells, HUH-7 2D, 3D HUH-7 organoid, and 3D Mixed organoids. Values represent relative gene expression mean ± SD. ^*^p < 0.05, ^**^p < 0.01, and ^***^p < 0.001 respectively

To investigate the molecular signature of the generated models and their relevance to specific HCC stages we evaluated a panel of differentially expressed genes reported to be expressed in specific HCC stages. These include stage I-specific genes (CA9, WNT7B), stage II-specific genes (APOBEC3B, FAM186A), two statistically significant differentially expressed genes in stage- III, DLG5, NCAPG2, and stage IV- specific gene GABRD [32]. Significant upregulation in APOBEC3B and CA9 genes was reported in 3D HUH-7 organoids (*p* < 0.01, 0.001) and 3D Mixed organoids (*p* < 0.01, 0.001) compared with HUH-7 cells. Also, DLG5 was significantly upregulated in the 3D HUH-7 organoids. WNT78 was upregulated in 3D HUH-7 organoids, but significantly downregulation in 3D Mixed counterpart (*p* < 0.05). Both 3D HUH-7 and 3D Mixed organoids showed significant downregulation in FAM186 and NCAPG2 compared with HUH-7 cells (Fig. 7A). Hierarchical clustering to the heatmap based on the complete-linkage method together with the Euclidean distance measure was used. Data shows that 3D Mixed organoids are closer in hierarchy to the HUH-7 cell line - originally generated from hepatoma tissue isolated from late-stage HCC patient [33]- than the 3D HUH-7 organoids (Fig. 7B).

## Discussion

Although 2D cultures of cell lines are regularly used in cancer research modeling, they fail to replicate the particularly complex tumor components. In this work, we report a promising organoid model for HCC characterized by a novel biomimetic matrix derived from human plasma and comprise both parenchymal and non-parenchymal cancer microenvironment elements. ECM is an integral compartment in the organotypic culture. It forms the niche in which cells reside and obtain critical biomechanical cues to support cell growth, proliferation, differentiation, and interaction with other components. These cues include growth factors, better access to nutrients and physical characteristics and are essential for determining the cell fate. Any subtle modification in these cues significantly influences cell behavior and is reflected in disease progression or resolution [34, 35]. Many factors influence the types of matrices currently used for cell cultures, such as safety, efficacy, biocompatibility, availability, and cost. ECM derived from animal sources is regularly used in the organotypic culture. However, their sources limit their reproducibility and translational and clinical applications because of residual genetic materials [36, 37]. For example, Matrigel is derived from mouse tumors, which are known for their heterogeneity. There is a lack of information regarding the composition, cellularity, or ECM remodeling within the tumor itself, making Matrigel an uncontrolled and randomized substrate extracted from animal tissue [38]. Platelet-rich plasma has been used for therapeutic applications in many areas of regenerative medicine, for example, regenerating damaged tissues [39] and endodontic and surgical periapical lesions [40, 41]. This role is attributed to their high content of growth factors and protein-rich composition [42]. PRP growth factors include epidermal growth factor (EGF), platelet-derived growth factor (PDGF), transforming growth factor-beta (TGF-beta), vascular endothelial growth factor (VEGF), fibroblast growth factor (FGF), insulin-like growth factor (IGF), and keratinocyte growth factor (KGF). PRP was reported to provide a bioactive scaffold that effectively stimulated chondrogenic differentiation of adipose and bone marrow-derived mesenchymal stromal cells, thanks to endogenous growth factors release [43, 44]. PRP was also used for engineering cartilage tissue that enhances pro-repair properties [45, 46]. It has a unique advantage of being applied as a personalized scaffold for clinically applied organoid culture systems. Unlike animal derived ECMs, it provides an autologous scaffold with zero non-human residuals. Standardization of PRP can follow several published protocols [47, 48]. As such, and compared to Matrigel, PRP offers a more relevant, personalized, more consistent, and well-characterized scaffold for organoid culture. Liver cancer organoids are promising tools for studying liver cancer and developing new treatments. Current liver cancer organoid models can recapitulate many of the features of human liver cancer, including heterogeneity, drug resistance, and metastatic potential. Organoids can also be used to model different subtypes of liver cancer, such as HCC and cholangiocarcinoma. However, there are still some challenges associated with liver cancer organoid models. These include the difficulty of generating and maintaining the organoids, and the lack of resemblance to the complex tumor microenvironment specific to liver cancer patients. Technical difficulties in developing cancer organoids involve the need for specialized skills and equipment, while biological issues arise due to variations in cell sources and culture conditions, and biocompatible supporting ECM.

In our model, we employed the HCC cell line HUH-7, and the stromal BM-MSCs and HUVECs grown in plasma-derived ECM to generate a reliable HCC organoid model. Previous reports by Isobe et al. [49] and Kitamura et al. [50] described the structure and cross-linkage density of fibrin clots generated from platelet-poor plasma. PRP-ECM provides a nutrient-rich, porous network structure to support free cell growth and communication. The porous microstructure and the high content of nutritive growth factors such as TGF$\beta$ and PDGF [51] enhanced the ability of the fibrin scaffold to support cell growth and survival [52–54]. Our data showed that PRP-ECM supported the development and assembly of both the 3D HUH-7 cells and the 3D Mixed organoids. The organotypic culture of HUH-7 alone in this new culture condition showed significantly different cancer properties and invasion potential compared to the 2D cultured cells. This can be attributed to the nutritive factors in the ECM conducive to self-assembly and cell communication. Subcutaneous transplantation of 3D HUH-7 organoids for 4 weeks in immunodeficient mice led to forming a sheet of malignant cells with adipocytes, hair follicles, and fine capillaries, further confirming the supportive function of the ECM matrix for cancer organoid engineering. In addition to its nutritive function, platelet-derived ECM also functioned as cell carrier and a vehicle for cell transplantation, maintaining the functionality and viability of the organoids.

Interestingly, the hypoxic necrotic core developed in the organoid may indeed mimic the solid tumor core, where uncontrolled cell growth and abnormal vascularization and metabolism result in a diminished supply of oxygen and nutrients. Hypoxic core may in fact promote cancer growth by means of a positive feedback loop that further enhances the cancer progression, and causes poor prognosis [55].

Including supportive stromal and endothelial cells with the cancer cells promotes dynamic interaction that mimics the native cancer niche and enhances cancer initiation, progression, metastasis, and acquired drug resistance [56]. This action is modulated via cytokine and proteases secretion, leading to modulating ECM remodeling, suppressing immune activity, and promoting angiogenesis and metastasis [57–61]. In the heterogeneous model (3D Mixed), we introduced the stromal and endothelial cells to highlight the crosstalk in the cancer environment and its impact on HCC behavior. In terms of structure, both the 3D HUH-7 and the 3D Mixed organoids grew and self-assembled freely into spherical structures within 14 days, similar to previous reports [62, 63]. However, introducing nonparenchymal cells formed a network or an outer layer surrounding the cancer cell core, as seen in the H&E-stained sections (Fig. 2). Importantly, these nonparenchymal cells induced the formation of a less necrotic core in the HCC mixed organoids. Propidium Iodide staining confirmed the retained viability of both organoids throughout the culture period and up to 21 days.

On the molecular level, the organotypic culture of HUH-7 organoids shifted the cell cycle toward higher expression of cyclin A and D and lower levels of cyclin E and B, showing more cells at the G2M phase [64–67]. Introducing the stromal compartment to the organoids raised the level of cyclin D expression and lowered the cyclin B expression suggesting rapid cell cycle progression [64–67]. Decreased apoptosis was observed in the organotypic culture of HUH-7 cells (HUH-7 organoids), in which APAF1, and P53 apoptosis inducers were downregulated [68, 69]. On the other hand, introducing the stromal and endothelial cells reduced the level of the cell cycle progression inhibitors P21 and BCL2 [70, 71], but restored APAF1 in both types of organoids. The 3D Mixed organoids maintained the closest molecular pattern to the HCC tissue in most cell cycle regulators and apoptosis-related markers, which may indicate a more relevant representation of the HCC cell behavior.

The invasion potential of the tumor cells is another determinant factor in the treatment and prognosis of cancer [72]. Recent studies have shown that stromal cells surrounding the cancer nest play a major role in progression and invasion [73, 74]. Degradation of the basement membrane via secretion of MMPs from both tumor and stromal cells contributes greatly to the ECM remodeling process and the loss of the migratory barriers toward a more invasive cancer phenotype [75–77]. Incorporating stromal cells significantly promoted the invasion of 3D Mixed organoids when compared to 3D HUH-7 ones, as shown by augmented expression of metalloproteases MMP-2, MMP-3, and MMP-13 and EMT markers, E-cadherin , Snail, and Vimentin. Interestingly, the organotypic culture of HUH-7 cells in PRP-ECM showed increased invasion markers compared to HUH-7 cells alone, supporting the prominent role of the ECM composition and culture system in modulating cell behavior [78]. Microscopic examination showed increased cellular invasion in the 3D Mixed organoids, further demonstrating more aggressive cancer properties.

Up-regulation of HCC genes c-Myc, TGF$\beta$, TCF4, RHOA, and IGF2 in the 3D Mixed organoids compared to the 3D HUH-7 ones suggests better modeling for increased cell proliferation and tumor growth leading to poor prognosis HCC tumors [79–83]. Aligned with the previous data, the genetic and protein expression levels of the widely known HCC marker AFP [84] were significantly higher in the 3D Mixed organoids when compared with the 3D HUH-7 organoids. In vivo transplantation however of the 3D Mixed organoids did not show similar malignant nodules to those of 3D HUH-7 organoids for the same time. This may indicate that the initial number of tumor cells, which is more in 3D HUH-7 organoids injected subcutaneously was a determining factor for tumor growth [85]. A prolonged transplantation time and a higher starting number of organoids may be required to evaluate the function of the 3D Mixed organoids *in vivo.*

Linear modeling analysis of gene expression across all HCC stages followed by several pairwise contrasts between the stages identified a set of differentially expressed genes (DEGs) for each stage [86]. We analyzed a set of stage-specific genes with high fold change to investigate the potential molecular signature of the developed model. Our data showed that the heterogeneous 3D Mixed organoid model displayed a gene expression pattern that is more relevant to advanced cancer stages compared to 3D HUH-7 organoid. The former model showed a lower expression level of APOBEC3H, DLG5, and FAM186A genes, which were reported to significantly increase in the early stages and decline later. The heterogeneous model had also a higher expression of CA9 gene compared to the 3D HUH-7 organoid, supporting a more advanced stage-relevant expression pattern, as CA9 was reported to be deferentially downregulated in the early stages and increased in later ones [87].

CSCs can self-renew and differentiate into cancer cell progeny and thus are believed to fuel aggressive cancer properties and acquired drug resistance in the tumor microenvironment [88]. CSC modeling is traditionally limited to 2D cell line culture, or tumor cells derived from primary cultures or differentiated from induced pluripotent stem cells. The inclusion of CSCs in organoid culture allows better analysis of CSCs in a more relevant microenvironment. Organotypic culture of both 3D HUH-7, and 3D Mixed organoid models showed a cell population with a phenotypic expression of CD24 and CD44, EPCAM, CD133. EPCAM is considered a key cancer stem cell regulator for cancer initiation and a marker for acquired stem cell criteria in tumor cells [89]. The HCC cell population that was positive for EPCAM and AFP was reported to display self-renewal and differentiation potential [90]. In another study, HUH-7 treatment with doxorubicin resulted in a significant increase in EpCAM/CD133 expression along with augmented stemness and tumor formation ability [91]. Cao et al. showed that HEPG2 tumorspheres expressed higher EpCAM, CD133, and CD44 levels and CSC-like features [92]. Cytotoxic drug resistance was assessed by analysis of side population assay after treatment with Cisplatin, which is commonly used by hepatic arterial infusion for HCC [93]. The heterogeneous 3D Mixed model showed a lower number of side population cells, which may contribute to drug resistance as reflected by the sustained viability and weak response to the apoptotic signals from BAX and P53 after doxorubicin treatment. This finding reflects the cell adhesion-mediated drug resistance mechanism, reported to be associated with the stromal cell compartment, which provided a kind of shield surrounding the cancer core and modulated the ECM remodeling leading to decreased chemotherapy efficacy [94].

The inclusion of the PRP-ECM in the organoids may also play a role in acquiring or maintaining cancer stem cell populations in the generated models, perhaps due to their rich content of PD-GF, bFGF, and TGF-β [42]. PDGF was reported to induce chemoresistance in ovarian cancer [95] and maintain stem cell criteria in glioma [96]. Also, bFGF and TGF-$\beta$ were reported to maintain stemness in lung cancer [97] and colon cancer [98].

In conclusion, cancer cell behavior and progression are determined based on several factors, including the cellular structure of the construct and the culture method [99]. Heterogeneous cell organoids are superior to single cell culture, mimicking the natural cancer microenvironment and providing a platform that facilitates studying the complex, realistic features in the tumor that contribute to cancer aggressiveness and spread. This model employed PRP as an optimum human natural derived ECM that can provide the organoids with optimum growth factors, chemicals, and the necessary physical cues for cancer growth and assembly within a heterogenous cancer microenvironment. Stromal cell compartments, such as stromal and endothelial cells, play an essential role in the cancer progression and response to treatment, and contribute to a relevant HCC cancer platform to study the intracellular interaction in chemotherapeutics testing. The present model and culture protocol could advance toward a more standardized and HCC-relevant HUH-7-derived organoid model. Eventually, these organoid models can be subjected to further expansion by adding an immune compartment to better delineate the role of the immune cells in tumor pathology and progression. Furthermore, future studies aim to standardize and testing of organoid models using various chemotherapies and treatment protocols over different time periods.

## Data Availability

Data sharing not applicable to this article as no datasets were generated or analyzed during the current study.

## References

[1] Sung H (2021). Global cancer statistics 2020: GLOBOCAN estimates of incidence and mortality worldwide for 36 cancers in 185 countries. Cancer J Clin.

[2] Llovet JM (2021). Hepatocellular carcinoma. Nat Reviews Disease Primers.

[3] Sevic I, et al. The role of the tumor microenvironment in the development and progression of hepatocellular carcinoma. Exon; 2019. pp. 29–45. 31664802

[4] Yin Z (2019). Heterogeneity of cancer-associated fibroblasts and roles in the progression, prognosis, and therapy of hepatocellular carcinoma. J Hematol Oncol.

[5] Li L, Wang H (2016). Heterogeneity of liver cancer and personalized therapy. Cancer Lett.

[6] Runa F (2017). Tumor microenvironment heterogeneity: challenges and opportunities. Curr Mol Biology Rep.

[7] Khaoustov VI (1999). Induction of three-dimensional assembly of human liver cells by simulated microgravity. Vitro Cell Dev Biology-Animal.

[8] He C et al. Liver Organoids, Novel and Promising modalities for Exploring and Repairing Liver Injury. Stem Cell Reviews Rep, 2022: p. 1–13. 10.1007/s12015-022-10456-3 PMC9534590 36199007

[9] Tharehalli U, Svinarenko M, Lechel A. *Remodelling and improvements in organoid technology to study liver carcinogenesis in a dish* Stem cells international, 2019. 2019. 10.1155/2019/3831213 PMC6399527 30915124

[10] Tian H (2022). Biophysics role and Biomimetic Culture systems of ECM stiffness in Cancer EMT. Global Challenges.

[11] Vukicevic S (1992). Identification of multiple active growth factors in basement membrane Matrigel suggests caution in interpretation of cellular activity related to extracellular matrix components. Exp Cell Res.

[12] Talbot NC, Caperna TJ (2015). Proteome array identification of bioactive soluble proteins/peptides in Matrigel: relevance to stem cell responses. Cytotechnology.

[13] Al Hrout A (2022). Modelling liver cancer microenvironment using a novel 3D culture system. Sci Rep.

[14] Sorrentino G (2020). Mechano-modulatory synthetic niches for liver organoid derivation. Nat Commun.

[15] Nguyen EH (2017). Versatile synthetic alternatives to Matrigel for vascular toxicity screening and stem cell expansion. Nat Biomedical Eng.

[16] Burnouf T (2013). Blood-derived biomaterials and platelet growth factors in regenerative medicine. Blood Rev.

[17] Song R et al. *Current development of biodegradable polymeric materials for biomedical applications* Drug design, development and therapy, 2018: pp. 3117–3145. 10.2147/DDDT.S165440 PMC6161720 30288019

[18] Spotnitz WD. *Fibrin sealant: the only approved hemostat, sealant, and adhesive—a laboratory and clinical perspective* International Scholarly Research Notices, 2014. 2014.

[19] Amable PR (2013). Platelet-rich plasma preparation for regenerative medicine: optimization and quantification of cytokines and growth factors. Stem Cell Res Ther.

[20] Weber M (2018). Blood-contacting biomaterials: in vitro evaluation of the hemocompatibility. Front Bioeng Biotechnol.

[21] Burnouf T (2009). Blood-derived biomaterials: fibrin sealant, platelet gel and platelet fibrin glue. ISBT Sci Ser.

[22] Nio K, Yamashita T, Kaneko S (2017). The evolving concept of liver cancer stem cells. Mol Cancer.

[23] Terris B, Cavard C, Perret C (2010). EpCAM, a new marker for cancer stem cells in hepatocellular carcinoma. J Hepatol.

[24] Zhuo J-Y (2020). CK19-positive hepatocellular carcinoma is a characteristic subtype. J Cancer.

[25] Uenishi T (2003). Cytokeratin 19 expression in hepatocellular carcinoma predicts early postoperative recurrence. Cancer Sci.

[26] Yeo W (2005). A randomized phase III study of doxorubicin versus cisplatin/interferon α-2b/doxorubicin/fluorouracil (PIAF) combination chemotherapy for unresectable hepatocellular carcinoma. J Natl Cancer Inst.

[27] Mazzocchi AR (2018). In vitro patient-derived 3D mesothelioma tumor organoids facilitate patient-centric therapeutic screening. Sci Rep.

[28] Lai CL (1988). Doxorubicin versus no antitumor therapy in inoperable hepatocellular carcinoma. A prospective randomized trial. Cancer.

[29] Abou-Alfa GK (2010). Doxorubicin plus Sorafenib vs doxorubicin alone in patients with advanced hepatocellular carcinoma: a randomized trial. JAMA.

[30] Baxter-Holland M, Dass CR (2018). Doxorubicin, mesenchymal stem cell toxicity and antitumour activity: implications for clinical use. J Pharm Pharmacol.

[31] Ma S (2008). CD133 + HCC cancer stem cells confer chemoresistance by preferential expression of the Akt/PKB survival pathway. Oncogene.

[32] Sarathi A, Palaniappan A (2019). Novel significant stage-specific differentially expressed genes in hepatocellular carcinoma. BMC Cancer.

[33] Nakabayashi H (1982). Growth of human hepatoma cell lines with differentiated functions in chemically defined medium. Cancer Res.

[34] Filipe EC, Chitty JL, Cox TR (2018). Charting the unexplored extracellular matrix in cancer. Int J Exp Pathol.

[35] Cox TR, Erler JT (2011). Remodeling and homeostasis of the extracellular matrix: implications for fibrotic diseases and cancer. Dis Models Mech.

[36] Urbischek M (2019). Organoid culture media formulated with growth factors of defined cellular activity. Sci Rep.

[37] Poudel H (2021). Synthetic matrices for Intestinal Organoid Culture: implications for Better Performance. ACS Omega.

[38] Kleinman HK, Martin GR. *Matrigel: basement membrane matrix with biological activity*. Elsevier. 10.1016/j.semcancer.2005.05.004 15975825

[39] Gutiérrez IQ, Sábado-Bundó H, Gay-Escoda C (2022). Intraarticular injections of platelet rich plasma and plasma rich in growth factors with arthrocenthesis or arthroscopy in the treatment of temporomandibular joint disorders: a systematic review. J Stomatology Oral Maxillofacial Surg.

[40] Gutiérrez IQ, Sábado-Bundó H, Gay-Escoda C. Intraarticular injections of platelet rich plasma and plasma rich in growth factors with arthrocenthesis or arthroscopy in the treatment of temporomandibular joint disorders: a systematic review. Journal of Stomatology, Oral and Maxillofacial Surgery; 2021. 10.1016/j.jormas.2021.12.006 34906730

[41] Del Fabbro M, Bortolin M, Taschieri S (2011). Is autologous platelet concentrate beneficial for post-extraction socket healing? A systematic review. Int J Oral Maxillofac Surg.

[42] Zoltowska A (2021). Plasma rich in growth factors in the treatment of endodontic periapical lesions in adult patients: a narrative review. Pharmaceuticals.

[43] Van Pham P (2013). Activated platelet-rich plasma improves adipose-derived stem cell transplantation efficiency in injured articular cartilage. Stem Cell Res Ther.

[44] Xie X (2012). Comparative evaluation of MSCs from bone marrow and adipose tissue seeded in PRP-derived scaffold for cartilage regeneration. Biomaterials.

[45] Petrera M (2013). Supplementation with platelet-rich plasma improves the in vitro formation of tissue-engineered cartilage with enhanced mechanical properties. Arthroscopy: J Arthroscopic Relat Surg.

[46] Ra Hara G, Basu T (2014). Platelet-rich plasma in regenerative medicine. Biomedical Res Therapy.

[47] Dhurat R, Sukesh MS (2014). Principles and methods of preparation of platelet-rich plasma: a review and author’s perspective. J Cutan Aesthetic Surg.

[48] Dashore S (2021). Preparation of platelet-rich plasma: National IADVL PRP taskforce recommendations. Indian Dermatology Online J.

[49] Isobe K (2017). Mechanical and degradation properties of advanced platelet-rich fibrin (A-PRF), concentrated growth factors (CGF), and platelet-poor plasma-derived fibrin (PPTF). Int J Implant Dentistry.

[50] Kitamura Y (2018). Platelet counts in insoluble platelet-rich fibrin clots: a direct method for accurate determination. Front Bioeng Biotechnol.

[51] Nishimoto S (2015). Growth factor measurement and histological analysis in platelet rich fibrin: a pilot study. J Oral Maxillofac Surg.

[52] Zhang Y, Alexander PB, Wang X-F (2017). TGF-β family signaling in the control of cell proliferation and survival. Cold Spring Harb Perspect Biol.

[53] Yokota J (2014). PDGF-induced PI3K-mediated signaling enhances the TGF–β–induced osteogenic differentiation of human mesenchymal stem cells in a TGF-β-activated MEK-dependent manner. Int J Mol Med.

[54] Ng F (2008). PDGF, TGF-β, and FGF signaling is important for differentiation and growth of mesenchymal stem cells (MSCs): transcriptional profiling can identify markers and signaling pathways important in differentiation of MSCs into adipogenic, chondrogenic, and osteogenic lineages. Blood J Am Soc Hematol.

[55] Muz B (2015). The role of hypoxia in cancer progression, angiogenesis, metastasis, and resistance to therapy. Hypoxia.

[56] Bussard KM (2016). Tumor-associated stromal cells as key contributors to the tumor microenvironment. Breast Cancer Res.

[57] Yang D et al. Role of endothelial cells in tumor microenvironment. Clin Translational Med, 2021. 11(6). 10.1002/ctm2.450 PMC8214858 34185401

[58] Guo S, Deng C-X (2018). Effect of stromal cells in tumor microenvironment on metastasis initiation. Int J Biol Sci.

[59] Goel S (2011). Normalization of the vasculature for treatment of cancer and other diseases. Physiol Rev.

[60] Klopp AH (2011). Concise review: dissecting a discrepancy in the literature: do mesenchymal stem cells support or suppress tumor growth?. Stem Cells.

[61] Kidd S (2009). Direct evidence of mesenchymal stem cell tropism for tumor and wounding microenvironments using in vivo bioluminescent imaging. Stem Cells.

[62] Sun L (2019). Modelling liver cancer initiation with organoids derived from directly reprogrammed human hepatocytes. Nat Cell Biol.

[63] O Oz, et al. 3d organoid modelling of hepatoblast-like and mesenchymal-like hepatocellular carcinoma cell lines. Hepatoma Res. 2021;7:60.

[64] Yang K, Hitomi M, Stacey DW (2006). Variations in cyclin D1 levels through the cell cycle determine the proliferative fate of a cell. Cell Div.

[65] Yang R (1999). Functions of cyclin A1 in the cell cycle and its interactions with transcription factor E2F-1 and the rb family of proteins. Mol Cell Biol.

[66] Siu KT, Rosner MR, Minella AC (2012). An integrated view of cyclin E function and regulation. Cell Cycle.

[67] Gavet O, Pines J (2010). Progressive activation of CyclinB1-Cdk1 coordinates entry to mitosis. Dev Cell.

[68] Aubrey BJ (2018). How does p53 induce apoptosis and how does this relate to p53-mediated tumour suppression?. Cell Death Differ.

[69] Cregan SP (2002). Apoptosis-inducing factor is involved in the regulation of caspase-independent neuronal cell death. J Cell Biol.

[70] Bélanger S (2005). Bcl-2 decreases cell proliferation and promotes accumulation of cells in S phase without affecting the rate of apoptosis in human ovarian carcinoma cells. Gynecol Oncol.

[71] Dash BC, El-Deiry WS (2005). Phosphorylation of p21 in G2/M promotes cyclin B-Cdc2 kinase activity. Mol Cell Biol.

[72] Wang W (2021). The clinical significance of microvascular invasion in the surgical planning and postoperative sequential treatment in hepatocellular carcinoma. Sci Rep.

[73] Papaccio F (2021). Profiling cancer-associated fibroblasts in melanoma. Int J Mol Sci.

[74] Hu B (2012). Multifocal epithelial tumors and field cancerization from loss of mesenchymal CSL signaling. Cell.

[75] Truong D et al. *Breast cancer cell invasion into a three dimensional tumor-stroma microenvironment* 2016. 6(1): pp. 1–18. 10.1038/srep34094 PMC5039718 27678304

[76] Chabottaux V et al. Membrane-type 4 matrix metalloproteinase (MT4‐MMP) induces lung metastasis by alteration of primary breast tumour vascular architecture. 2009. 13(9b): p. 4002–13. 10.1111/j.1582-4934.2009.00764.x PMC4516547 19426156

[77] Winkler J (2020). Concepts of extracellular matrix remodelling in tumour progression and metastasis. Nat Commun.

[78] Lu P, Weaver VM, Werb Z (2012). The extracellular matrix: a dynamic niche in cancer progression. J Cell Biol.

[79] Yuen MF et al. Expression of c-Myc, c‐Fos, and c‐jun in hepatocellular carcinoma. 2001. 91(1): p. 106–12. 10.1002/1097-0142(20010101)91:1<106::aid-cncr14>3.0.co;2-2 11148566

[80] Zhou L, Liu J (2006). J.W.j.o.g.W. Luo. Serum Tumor Markers Detect Hepatocellular Carcinoma.

[81] Teng K et al. KIFC1 is activated by TCF-4 and promotes hepatocellular carcinoma pathogenesis by regulating HMGA1 transcriptional activity. 2019. 38(1): p. 1–20. 10.1186/s13046-019-1331-8 PMC6657086 31340839

[82] Bai Y et al. The diagnostic and prognostic role of RhoA in hepatocellular carcinoma. 2019. 11(14): p. 5158. 10.18632/aging.102110 PMC6682515 31339860

[83] Martinez-Quetglas I et al. IGF2 is up-regulated by epigenetic mechanisms in hepatocellular carcinomas and is an actionable oncogene product in experimental models. 2016. 151(6): p. 1192–205. 10.1053/j.gastro.2016.09.001 PMC12951732 27614046

[84] Debruyne EN. and J.R.J.C.c.a. Delanghe, *Diagnosing and monitoring hepatocellular carcinoma with alpha-fetoprotein: new aspects and applications*. 2008. 395(1–2): p. 19–26. 10.1016/j.cca.2008.05.010 18538135

[85] Hoffmann B et al. The initial engraftment of tumor cells is critical for the future growth pattern: a mathematical study based on simulations and animal experiments. 2020. 20(1): p. 1–14. 10.1186/s12885-020-07015-9 PMC7275472 32503458

[86] Sarathi A. and A.J.B.c. Palaniappan, *Novel significant stage-specific differentially expressed genes in hepatocellular carcinoma*. 2019. 19(1): p. 1–22. 10.1186/s12885-019-5838-3 PMC6612102 31277598

[87] Nakabayashi H (1982). Growth Hum Hepatoma cell Lines Differentiated Funct Chemically Defined Medium.

[88] Shimono Y, et al. *Organoid culture of human cancer stem cells*, in *Organoids*. Springer; 2016. pp. 23–31. 10.1007/7651_2016_13 27654995

[89] Han J (2020). Cancer stem cell-targeted bio-imaging and chemotherapeutic perspective. Chem Soc Rev.

[90] Yamashita T (2009). EpCAM-positive hepatocellular carcinoma cells are tumor-initiating cells with stem/progenitor cell features. Gastroenterology.

[91] Karabicici M (2021). Doxorubicin-induced senescence promotes stemness and tumorigenicity in EpCAM–/CD133 – nonstem cell population in hepatocellular carcinoma cell line, HuH‐7. Mol Oncol.

[92] Nygaard V (2003). Effects of mRNA amplification on gene expression ratios in cDNA experiments estimated by analysis of variance. BMC Genomics.

[93] Osaki A (2013). A safe and effective dose of cisplatin in hepatic arterial infusion chemotherapy for hepatocellular carcinoma. Cancer Med.

[94] Hazlehurst LA, Dalton WS (2001). Mechanisms associated with cell adhesion mediated drug resistance (CAM-DR) in hematopoietic malignancies. Cancer Metastasis Rev.

[95] Raghavan S (2020). Carcinoma-associated mesenchymal stem cells promote chemoresistance in ovarian cancer stem cells via PDGF signaling. Cancers.

[96] Kim Y (2012). Platelet-derived growth factor receptors differentially inform intertumoral and intratumoral heterogeneity. Genes Dev.

[97] Meng Y (2019). Basic fibroblast growth factor signalling regulates cancer stem cells in lung cancer A549 cells. J Pharm Pharmacol.

[98] Otte J (2019). FGF signalling in the self-renewal of colon cancer organoids. Sci Rep.

[99] Tang Y (2020). Autologous culture method improves retention of tumors’ native properties. Sci Rep.

